# Multi-System Physical Exercise Intervention for Fall Prevention and Quality of Life in Pre-Frail Older Adults: A Randomized Controlled Trial

**DOI:** 10.3390/ijerph17093102

**Published:** 2020-04-29

**Authors:** Jiraporn Chittrakul, Penprapa Siviroj, Somporn Sungkarat, Ratana Sapbamrer

**Affiliations:** 1 Department of Community Medicine, Faculty of Medicine, Chiang Mai University, Chiang Mai 50200, Thailand; jerasooutch@gmail.com (J.C.); lekratana56@yahoo.com (R.S.); 2 Department of Physical Therapy, Faculty of Associated Medical Sciences, Chiang Mai University, Chiang Mai 50200, Thailand; somporn.sungkarat@cmu.ac.th

**Keywords:** frailty, multi-system physical exercise, fall risk, quality of life, older adults

## Abstract

Effective interventions for indicated fall prevention are necessary for older adults with frailty. We aimed to determine the effectiveness of a Multi-system Physical Exercise (MPE) for fall prevention and Health-Related Quality of Life (HRQOL) in pre-frail older adults. This randomized control trial with allocation concealment included 72 adults aged 65 and above, identified as pre-frailty and with mild and moderate fall risk scores measured by the Physiological Profile Assessment (PPA). Randomly, using block randomization, participants were divided into two groups: an MPE group (n = 36) and a control group (n = 36). The intervention consisted mainly of proprioception, muscle strengthening, reaction time, and balance training and was carried out three days per week for 12 weeks. The primary outcome was fall risk assessed using PPA at 12 weeks post-baseline and at a 24 week follow-up. Significant differences were found in the improvement in fall risk, proprioception, muscle strength, reaction time and postural sway, and fear of fall scores in the MPE group compared with controls at week 12 and 24. In addition, HRQOL had increased significantly in the MPE group in comparison to controls. The MPE program significantly increased muscle strength and improved proprioception, reaction time, and postural sway leading to fall risk reduction in older adults with pre-frailty. Therefore, the MPE program is recommended for used in day-to-day primary care practice in the pre-frail population.

## 1. Introduction

The aging population constitutes a large and increasing percentage of the world population [1]. When people grow older, they experience a deterioration in their physiological system and functional status decline until they become frail [2,3]. Frailty is defined as an age-related decline of multiple physiological processes and functions, having a negative impact on multiple adverse health outcomes such as disability, injury, multiple-disease, hospitalization, illness, falls, and mortality [4]. In studies, 62.8% of older people were found to have one to two falls resulting in an increased risk of falls and consequent injuries [5,6,7]. In addition, falls were associated with a higher risk of future falls (OR: 1.84) [8]. Falling can lead to mortality, morbidity, suffering for older people and their families, and social costs associated with hospitalization [9,10,11]. Death in 84.6% of older adults is more likely to occur from fall-related injuries than from other causes, and 38.19% of hospital admissions are a consequence of fall injuries [12]. Fall prevention is important for frail older adults because it affects their well-being and the healthcare system.

Falls are a common problem for older people. A lack of exercise and malnutrition leads to a decrease in muscle strength and physical activities. These affect the strength, proprioception, balance, and functional capacity, which can cause falls in the elderly [13,14,15,16]. The first cause of falls is usually balance impairment; followed by a deterioration of the musculoskeletal system; the nervous system; sensory degradation. Vision is the second leading cause [16,17]. Exercise is key for the prevention of falls when compared to other treatments [18]. There are many exercise interventions for reducing falls. These include balance and flexibility training, a resistance exercise strengthening program, and endurance training, all of which have been shown to be effective in improving physical fitness and reducing falls in the community-dwelling elderly [14,19,20,21,22,23,24].

There is strong evidence to show multifactorial exercise intervention prevents falls in community-dwelling older adults [25]. A previous study found that multifactorial intervention improves performance in reducing risk factors of falls in frail older people. However, this study involved an individualized home exercise program and focused only on balance and lower limb strength training. The program was managed by physiotherapists with interdisciplinary management of medical, psychological, and social problems [26]. There are very few studies into the efficacy of exercise intervention designed to address the physiological problems related to falls, and the effect of exercise interventions on falls in frail older adults is unclear. Fall prevention necessitates early intervention in pre-frail older adults to improve the physical performance as it can reverse the frailty level and reduce the risk of falls. In addition, previous studies found that exercise can improve depression [27] and Health-Related Quality of Life (HRQOL) in frail and pre-frail older adults [28]. Our intervention, Multi-system Physical Exercise (MPE), includes four components for reducing the risk of falls in older adults, specifically proprioception training, muscle strength training, reaction time exercise training, and postural balance training. This study used the Physiological Profile Assessment (PPA), a valid and reliable measurement to assess the effectiveness of this intervention [29]. In summary, the aim of this study was to determine the effectiveness of this intervention on fall prevention, depression, and Health-Related Quality of Life (HRQOL) in community-dwelling older adults with pre-frailty.

## 2. Materials and Methods

### 2.1. Study Design

This study was designed as a single-blind (assessor-blind), stratified randomized trial controlled by sex and age with a concealed allocation and intention-to-treat analysis. The trial was registered at the Thai Clinical Trial Register: Identification number TCTR20180806001. Pre-frail older adults registered in the primary care center were randomly divided into two groups, the Multi-system Physical Exercise (MPE) group and stretching exercise (control) group.

### 2.2. Participants

**Figure 1:**
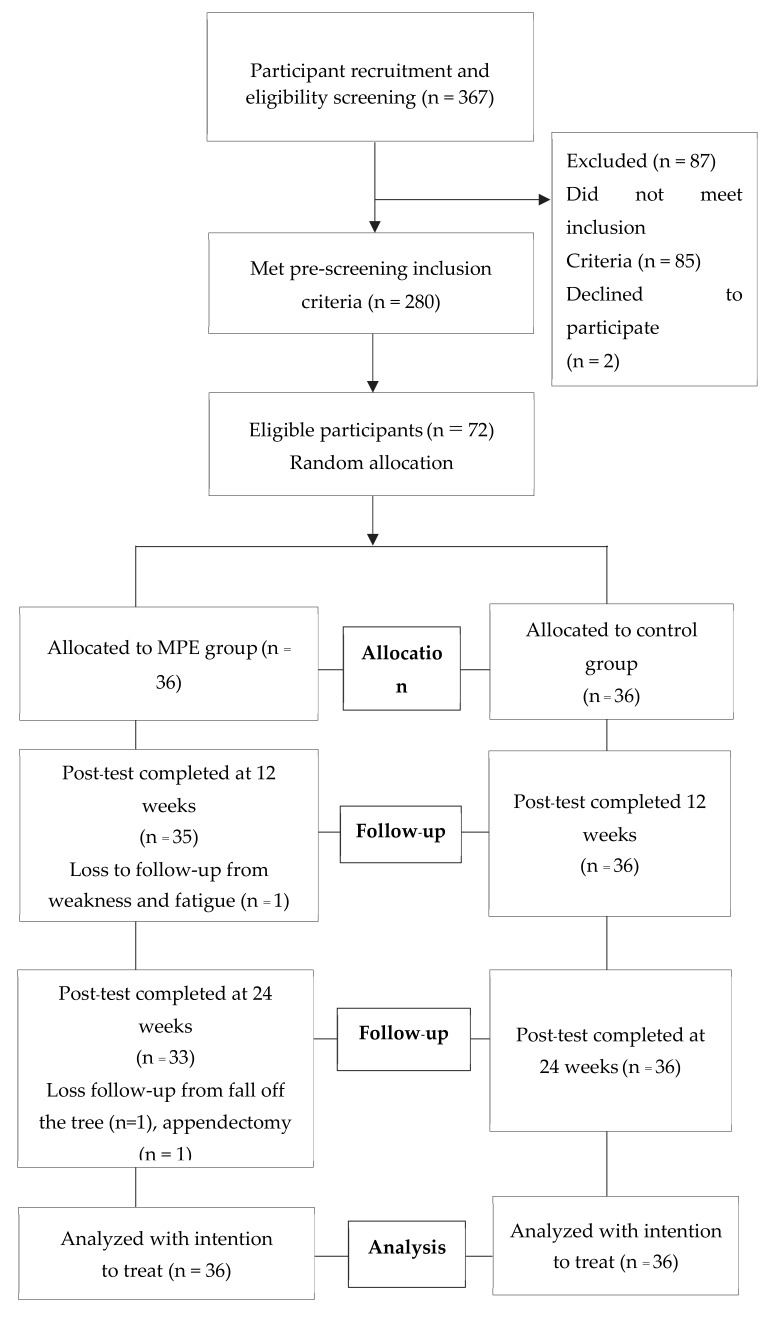
The flow diagram of the participants through each stage of the study.

As shown in Figure 1, a total of 367 older adults were assessed and consented to participate in the present study (87 of these did not meet the eligibility criteria). Participants were recruited from the older population identified from the management records of the primary care center. The inclusion criteria were as follows: (1) older adults, aged 65 years or above; (2) had been identified as pre-frail according to Fried’s Frailty Phenotype [30]; and (3) were at risk of falling using PPA composite at zero points and above [29]. The older adults were excluded if they met any of the following criteria: receiving palliative care; having a diagnosis of heart disease, cancer, and severe depression; or taking psychotropic, antiarrhythmic, or hypnotic drugs. Of this group, 72 participants were randomly selected and then randomly assigned to MPE (n = 36) and control (n = 36) groups using block randomization. Each stratum was allocated to blocks of four and randomized by drawing lots. Pre- and post-intervention, all assessments were evaluated by the researcher who was blind to the program at baseline and weeks 12 and 24.

The sample size was based on an estimated minimally required sample size for analysis. G*power software version 3.0 was used, and 33 participants per group were needed for a two-tailed at 5% significance level, anticipated effect size = 0.71 [31], with a statistical power level of 80%. Allowing for a 10% dropout rate, 36 subjects were recruited into the study.

### 2.3. Intervention

The intervention group participated in a Multi-system Physical Exercise (MPE) program that was designed based on the components of a fall risk assessment using the Physiological Profile Assessment (PPA) [29] and literature concerning exercise interventions for fall prevention [21]. The MPE program consisted of four parts: proprioception training, muscle strength training, reaction time exercise training with auditory cues, and postural balance training (see Table 1).

The MPE program carried out with the participants in the intervention group for three days per week for 12 weeks, totaling 36 sessions. To ensure that all participants exercised correctly and safely, they exercised in a supervised sub-group of twelve participants. Each training session was booked for 60 min, starting with a ten minute warm-up, and ending with a five minute cool-down. All participants began the program by learning the basics of the four components of the program. The program was divided into three levels, beginner, intermediate, and advanced, however all participants in this study had comparable ability at baseline, therefore, all participants were started at the beginner level then moved to intermediate and advanced levels as a group. The exercise regimens were geared to enable people to pass each level. Each component of exercise had three sets, each carried out for 15 repetitions, and participants were instructed to maintain the contraction for 10 s. Rest intervals were set as 10 s between each set. A lead instructor who is a physiotherapist with experience in teaching exercises delivered the 12 week training course. The control group received the flexibility exercise training three times each week of the program. They met a researcher at the primary care unit once a week for the 12 consecutive weeks of the study to share their health experience.

### 2.4. Outcome Measures

#### 2.4.1. Primary Outcome

**Figure 2:**
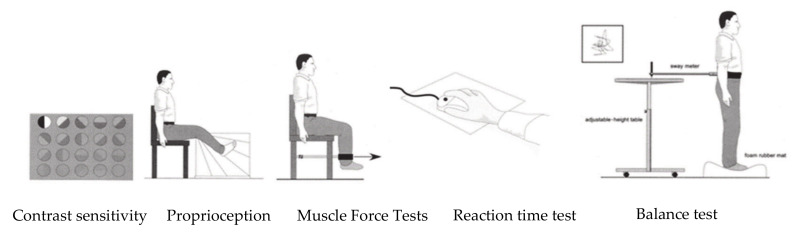
Physiological Profile Assessment (PPA) for fall risk scores template modified from [29].

Fall risk was measured using the Physiological Profile Assessment (PPA) for fall risk [29]. The PPA has been shown to provide valid and reliable measurements for assessing fall risk and evaluating the effectiveness of interventions. PPA involves a series of simple tests of vision, peripheral sensation, muscle force, reaction time, and postural sway. These were assessed using the following: (1) vision test (Edge-contrast sensitivity) using the Melbourne Edge test; (2) peripheral sensation (proprioception) test using a lower limb–matching task; (3) muscle force taken as the maximal voluntary strength of the knee extensor muscle in the subjects’ dominant (stronger) leg measured under isometric conditions in a seated position; (4) reaction time using hand reaction, which was recorded by time and (5) postural sway using a sway meter that measures displacement of the body (see Figure 2). All five parts of PPA fall risk were measured by a physiotherapist. NeuRA FallScreen^®^ software was used to calculate the PPA fall risk score [29].

#### 2.4.2. Secondary Outcomes

The fear of falling was measured using the Thai Fall Efficacy Scale—International questionnaire, which has a Cronbach’s alpha coefficient of 0.95 [32]. Depression was assessed using the Thai Geriatric Depression Scale (TGDS), which has a Cronbach’s alpha coefficient of 0.85 [33]. The TGDS has a total score of 0–15 points, with 15 questions to assess the feelings of the subject during the past week. The recommended cutoff scores of the TGDS are similar to those of the English version, which are 0–4 points for no depression, 5–10 points for suggestive depression, and 11–15 points for indicative depression [33,34]. Health-Related Quality of Life (HRQOL) was assessed using the 36-Item Short-Form Health Survey questionnaire (SF-36) Thai version. It was found that the reliability was 0.7 (0.72–0.86) across all dimensions. The SF-36 is categorized into two main components, physical and mental, with a total of 11 main questions [35,36]. The overall scores range from 0–100, with higher scores indicating better HRQOL.

### 2.5. Data Analysis

The data were analyzed using SPSS version 22. The Shapiro–Wilk test was used to check normality. Values were expressed as mean ± Standard Deviation (SD) for continuous data, and frequencies were presented in the case of categorical variables. Baseline characteristics were compared with all outcomes between the two groups using an independent sample *t*-test. The comparison of all outcomes between baseline, week 12, and week 24 was carried out using the two-way repeated measures ANOVA. The analysis was carried out using the intention-to-treat approach, with missing data handled using the last observation carried forward method. The *p*-value was deemed statistically significant at 0.05 (two-tailed).

### 2.6. Ethical Consideration

All participants provided written informed consent to participate in this study. The study was conducted in accordance with the Declaration of Helsinki, and the protocol was approved by the Research Ethics Committee of the Faculty of Medicine, Chiang Mai University, Thailand (no. 187/2018).

## 3. Results

### 3.1. Baseline Characteristics

The total number of subjects was 72 (intervention group = 36, control group = 36) at baseline. There was no statistically significant difference between the two groups regarding age, average number of comorbidities and number of drugs, body mass index, and fall risk score (Table 2).

### 3.2. Fall Risk Score

**Figure 3:**
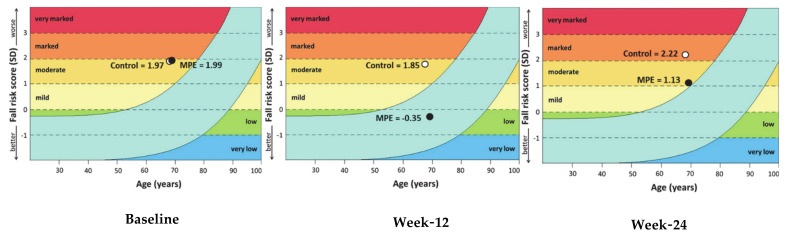
The graphs are defined by the mean fall risk score tested. The *X*-axis is the average age, and *Y*-axis is the fall risk. The light blue curved band shows the normal range. The two lines show the 75th percentile (top-line) and the 10th percentile (bottom-line) of the risk of falling.

Baselines of fall risk scores between MPE and control groups were not significantly different. Post-intervention at week 12 and 24, there was a statistically significant difference in fall risk scores between the MPE and control groups at weeks 12 and 24 (*p* < 0.001) (Table 3). Moreover, Figure 3 shows that fall risk scores in both groups were a moderate risk level at baseline. After 12 weeks of intervention it was found that the MPE group had a low-risk level, whereas people in the control group were at moderate risk. After 24 weeks of intervention, we found the control group were a marked risk but the intervention group were at moderate risk at week 24.

There was no statistically significant difference in edge-contrast sensitivity (dB) between the two groups. There was also no significant difference in edge-contrast sensitivity (dB) between pre- and post-intervention between the MPE and control groups. Baselines of proprioception, knee extension strength (kg force), hand reaction time (ms), and postural sway (mm^2^) between the MPE and control groups were not significantly different. However post-intervention at weeks 12 and 24, there was a statistically significant difference in proprioception, knee extension strength (kg force), hand reaction time (ms), and postural sway (mm^2^) between the MPE and control groups at weeks 12 and 24 (*p* < 0.001 and *p* < 0.05) (Table 3).

#### 3.2.1. Fear of Falling Scores

At baseline, fear of falling scores between the intervention and control groups were not significantly different. Post-intervention at weeks 12 and 24, there was a statistically significant decrease in the score between the two groups (*p* < 0.001) (Table 4).

#### 3.2.2. Depression Scores

Baselines depression scores between the intervention and control groups were not significantly different. At week 12 post-intervention, there was a statistically significant difference in the decrease in depression scores between the two groups (*p* = 0.001), however, at week 24, there were no significant differences between the two groups (Table 4). The TGDS scores indicated that all participants in both MPE and control groups did not have depressive symptoms from baseline throughout the 24 week follow-up period.

#### 3.2.3. Health-Related Quality of Life (HRQOL)

The baselines of the overall HRQOL between MPE and control groups were significantly different (*p* < 0.001). At week 12, there was a statistically significant difference in the overall HRQOL between the MPE and control groups (*p* < 0.05). Post-intervention at week 24, there were no significant differences between the two groups (Table 4).

## 4. Discussion

Pre-frail is the frailty status that signifies a degenerative physiological system, which indicates a potentially high risk of falling. This MPE program may be used to consequently reduce the fall risk. Our results showed that the 12 week Multi-system Physical Exercise (MPE) program had a significant effect in improving proprioception, reaction time, knee extension, and balance in the pre-frail adults in the study, which reflected improved overall physical performance and reduced fall risk. The effects of these outcome improvements were sustained until the end of the 24th week. However, after intervention at week 24, we found that the control group had increased fall risk scores from both the baseline and week 12. There may be many factors causing these such as lack of exercise, ill health, environmental factors, social factors, and personal factors [13]. Our results are consistent with previous studies, which indicated that exercise intervention had beneficial effects on improving physical performance and reducing fall risk in the frail and pre-frail elderly living in community [38,39,40]. Exercise intervention has been recommended to improve muscle strength, balance, and gait ability to reduce the risk of falls [41]. Nevertheless, the effect on edge-contrast sensitivity (dB) was not significantly different between the two groups and was not different pre- and post-intervention because our program had no visual ability training. Further, the performance declines observed after the exercise cessation at week 12 suggest the need for continuing exercise to maintain the beneficial effects of the MPE program.

It has already been reported in literature that high-frequency proprioceptive training can reduce the risk of falls in older adults [42]. A proprioception-training program for 12 weeks in older adults is effective in improving postural stability and decreasing the risk of falls [43]. Therefore, exercise can improve the somatosensory and sensorimotor function, which results in better proprioception [44,45]. However, there are few studies on proprioception exercise training in the pre-frail older adults. Our results showed that outcomes related to proprioception in older adults with pre-frailty were a significantly different between the intervention and control groups (*p* < 0.001).

The physiological changes in older adults with frailty have been related to decreased testosterone levels associated with the loss of muscle and strength and muscle atrophy [46,47,48,49]. Exercise can improve the muscle contractile protein synthetic pathways and increase muscle power, which can increase muscle strength [22,50]. With regard to exercise intervention, our results are consistent with a previous study that found that resistance and aerobic exercise intervention had beneficial effects on muscle strength [51,52] and elastic band exercise improves muscle strength in pre-frail older adults [53].

Additionally, our results indicated that the reaction times were significantly different (*p* < 0.001) between the intervention and control groups, which was consistent with a previous study that showed step training improved reaction time and reduced falls in the older adults (*p* < 0.001) [54]. Changes in response time at an older age for more complex response time tasks, such as jobs that require more complex motor processes, requiring fast reactions and accurate responses, are important for responding to dual tasks and also for walking. Both these areas, if improved can reduce the risk of falling [55,56]. Meanwhile, our results found that the MPE program especially the balancing exercise, helps train the center of pressure and center of mass that can improve postural sway and stability therefore reducing the fall risk [57,58]. Our results are consistent with the results reported in the literature [18,59].

The fear of fall scale is one of the most commonly tools used to assess fall risk [60] originating from an individual appraisal of the abilities of the patient to maintain balance [61]. Our finding was that the fear of falls was significantly different between the intervention and control groups (*p* < 0.001). Consistent with previous studies, we found that exercise intervention could reduce the fear of falls in frail and pre-frail older adults [62]. Likewise, it was found that the MPE program significantly reduced the level of depression in comparison to the control group in the pre-frail older adults at week 12 (*p* = 0.001), whereas there was no significant difference in week 24. This finding is consistent with the results reported in other literature, which reported that exercise intervention significantly reduced depression in frail older adults [63].

It is unclear why the control group had a significantly better HRQOL score than the MPE group given that all their demographic characteristics and outcome measures were comparable at baseline. It is possible that factors not included in the study such as family care and financial status may account for such differences. Nevertheless, participants in the MPE program demonstrated significant improvement in the overall HRQOL when compared with control group at week 12 (*p* < 0.001). It has been suggested that exercise and physical activity significantly contributes to physical and mental well-being, which leads to an improved quality of life [64]. These results are consistent with previous studies, which reported that HRQOL was improved following exercise intervention in frail and pre-frail older adults [65,66].

The strength of this study is that a physical exercise program is designed to address the physiology of falls specific for pre-frail older adults. This study has several limitations that need to be considered. First, our exercise program is designed as group exercise, which makes it impossible to manage the intensity of exercise as appropriate for each individual. However, group exercise often results in a higher level of euphoria than does individual exercise [67]. In further studies, the physical fitness of each person should be assessed and the subjects should be arranged into groups with the same capability to carry out the exercise. Second, this program was designed as a center-based exercise program, making it difficult for subjects to perform at home. Therefore, a home-based program should be designed for continuous and effective use in the elderly.

## 5. Conclusions

The results indicated that Multi-system Physiological Exercise (MPE) has the potential for reducing the risk of falls, improving proprioception, hand reaction time, and sway path, and increasing the knee extension strength among community-dwelling older adults with pre-frailty. The intervention also leads to a decrease in the fear of falling, depression, and an increase in the quality of life. These results suggest that the MPE is a viable choice for fall prevention in the older adults in the community. Further research is required to determine the exact program required for specific exercise training to prevent falls in a specific population of older adults living with frailty or chronic diseases.

## Figures and Tables

**Table 1:** Multi-system Physical Exercise (MPE) training protocol.

**Beginner** **(Weeks 1 to 4)**	Intermediate (Weeks 5 to 8)	Advanced (Weeks 9 to 12)
**Proprioception** Seated ankle ball Single leg stance (hip flexion) with support and with flexion and extension of knees Single leg stance (hip extension) with support and with flexion and extension of knees	**Proprioception** Standing ankle ball with support Single leg stance (hip flexion) with eyes closed and with support and with flexion and extension of knees Single leg stance (hip extension) with eyes closed and with support and with flexion and extension of knees	**Proprioception** Standing ankle ball without support Single leg stance (hip flexion) without support and with flexion and extension of the knee Single leg stance (hip extension) without support and with flexion and extension of knees
**Muscle strengthening** Seated alternate double knee lifts with weights Modified chair stands (Challenger) Seated alternate kicks with weighHip extension standing with support with weights	**Muscle strengthening** Sit-to-stand with support Knee raise standing with support and with weight Squats to a chair with supportStep back lunges with support	**Muscle strengthening** Sit-to-stand without support Knee raise standing without support and with weight Squats to a chair without support Step back lunges without support
**Reaction time** Seated alternate touches to front Seated alternate touches to back Seated alternate touches to side Seated alternate touches to 3 steps (forward, side, backward)	**Reaction time** Step-up with support Step forward standing with support Step backward standing with support Step to sides standing with support 3 steps standing (forward, side, and backward) with support	**Reaction time** Step up without support Step forward standing without support Step backward standing without support Step to sides standing without support 3 steps standing (forward, side, and backward) without support
**Balance** Seated alternate weight shifts Seated calf and toe raise Heel raise with support Crossover with support	**Balance** Heel-to-toe standing with support Side leg raise with support Heel raises without support Calf and toe raise without support Crossover without support	**Balance** Heel-to-toe without support Leaning star Heel walking Toe walking 8 shaped walking flex mat

**Table 2:** Baseline characteristics of participants.

Characteristics	MPE Group (n = 36)	Control Group (n = 36)	*p*-Value
Age (years), mean ± SD	69.14 ± 3.55	68.89 ± 3.86	0.776
65–69, n (%)	24 (66.7)	23 (63.9)	
70–74, n (%)	8 (22.2)	9 (25)	
≥75, n (%)	4 (11.1)	4 (11.1)	
Number of comorbidities, mean ± SD	0.86 ± 0.76	0.83 ± 0.60	0.865
Number of drugs, mean ± SD	0.86 ± 0.76	0.77 ± 0.54	0.594
Body mass index ^≠^ (kg/m^2^), mean ± SD	24.46 ± 4.06	24.32 ± 4.36	0.883
Underweight <18.5, n (%)	2 (5.6)	4 (11.1)	
Normal weight 18.5–22.9, n (%)	12 (33.3)	9 (25)	
Overweight 23.0–27.5, n (%)	15 (41.7)	15 (41.7)	
Obese >27.5, n (%)	7 (19.4)	8 (22.2)	
Fall risk (Z-score), mean ± SD	1.99 ± 0.58	1.97 ± 0.61	0.862

Analyzed by independent sample *t*-test, ^≠^ Asian body mass index [37].

**Table 3:** Comparison of fall risk between intervention and control groups.

Outcomes	Baseline ^a^	Week 12 ^b^	Week 24 ^c^	*p*-Value Within Group
	Mean ± SD	Mean ± SD	Mean ± SD	
**Fall risk index score**				
MPE Group	1.99 ± 0.58	−0.35 ± 0.72	1.13 ± 0.84	<0.001 ^ab,ac,bc^
Control Group	1.97 ± 0.61	1.85 ± 0.59	2.22 ± 0.55	<0.05 ^ac,bc^
***p*-Value between Groups**	0.862	<0.001	<0.001	
**Edge-contrast sensitivity (dB)**				
MPE Group	21.13 ± 1.01	21.36 ± 1.15	21.27 ± 1.03	0.063
Control Group	21.05 ± 0.89	21.02 ± 0.81	20.97 ± 0.84	0.907
***p*-Value between Groups**	0.713	0.160	0.173	
**Proprioception (degree error)**				
MPE Group	2.77 ± 1.60	0.48 ± 1.13	1.56± 1.48	<0.001 ^ab,ac,bc^
Control Group	2.71 ± 1.44	3.13 ± 1.45	3.01 ± 1.73	0.383
***p*-Value between Groups**	0.878	<0.001	<0.001	
**Knee extension strength (kg force)**				
MPE Group	13.87 ± 5.17	18.75 ± 6.03	13.06 ± 5.58	<0.001 ^ab,bc^
Control Group	16.56 ± 7.31	13.20 ± 5.01	7.47 ± 4.85	<0.001 ^ab,ac,bc^
***p*-Value between Groups**	0.077	<0.001	<0.001	
**Hand reaction time (ms)**				
MPE Group	336.80 ± 67.91	251.98 ± 37.74	318.81 ± 60.57	<0.001 ^ab,bc^
Control Group	345.76 ± 79.26	355.08 ± 45.73	386.40 ± 66.42	0.002 ^ac,bc^
***p*-Value between Groups**	0.608	<0.001	<0.001	
**Sway path (mm^2^)**				
MPE Group	1642 ± 1038.05	452.41 ± 377.80	902.47 ± 473.75	<0.001 ^ab,ac,bc^
Control Group	1433.08 ± 754.49	1018.86 ± 351.66	1209.52 ± 401.39	0.001 ^ab,bc^
***p*-Value between Groups**	0.332	<0.001	0.004	

^ab^*p*-value of baseline compared with week 12, ^ac^*p*-value of baseline compared with week 24, ^bc^*p*-value of week 12 compared with week 24.

**Table 4:** Comparison of fear of falling, depression, and HRQOL between intervention and control groups.

Outcomes	Baseline ^a^	Week 12 ^b^	Week 24 ^c^	*p*-Value within Group
	Mean ± SD	Mean ± SD	Mean ± SD	
**Fear of Fall Score**				
MPE Group	40.13 ± 6.60	18.05 ± 4.85	24.27 ± 12.23	<0.001 ^ab,ac,bc^
Control Group	37.55 ± 13.00	25.69 ± 9.97	38.52 ± 12.47	<0.001 ^ab,bc^
***p*-Value between Groups**	0.292	<0.001	<0.001	
**Depression Score**				
MPE Group	2.77 ± 1.74	0.50 ± 0.87	2.58 ± 2.03	<0.001 ^ab,bc^
Control Group	2.58 ± 2.28	1.16 ±0.77	2.30 ± 1.60	<0.001 ^ab,bc^
***p*-Value between Groups**	0.686	0.001	0.522	
**Overall Health-Related Quality of Life (HRQOL)**				
MPE Group	67.35 ± 10.82	93.91 ± 9.00	85.62 ± 12.34	<0.001 ^ab,bc,ac^
Control Group	76.96 ± 8.66	90.42 ± 1.68	81.96 ± 7.86	<0.001 ^ab,bc,ac^
***p*-Value between Groups**	<0.001	0.025	0.138	

^ab^*p*-value of baseline compared with week 12, ^ac^*p*-value of baseline compared with week 24, ^bc^*p*-value of week 12 compared with week 24.
